# *In Vivo* Two Photon Imaging of Astrocytic Structure and Function in Alzheimer’s Disease

**DOI:** 10.3389/fnagi.2018.00219

**Published:** 2018-07-19

**Authors:** Patricia Kelly, Eloise Hudry, Steven S. Hou, Brian J. Bacskai

**Affiliations:** Massachusetts Institute for Neurodegenerative Disease, Department of Neurology, Massachusetts General Hospital, Harvard Medical School, Harvard University, Boston, MA, United States

**Keywords:** Alzheimer’s disease, astrocytes, two-photon microscopy, *in vivo* imaging, calcium

## Abstract

The physiological function of the neurovascular unit is critically dependent upon the complex structure and functions of astrocytes for optimal preservation of cerebral homeostasis. While it has been shown that astrocytes exhibit aberrant changes in both structure and function in transgenic murine models of Alzheimer’s disease (AD), it is not fully understood how this altered phenotype contributes to the pathogenesis of AD or whether this alteration predicts a therapeutic target in AD. The mechanisms underlying the spatiotemporal relationship between astrocytes, neurons and the vasculature in their orchestrated regulation of local cerebral flow in active brain regions has not been fully elucidated in brain physiology and in AD. As there is an incredible urgency to identify therapeutic targets that are well-tolerated and efficacious in protecting the brain against the pathological impact of AD, here we use the current body of literature to evaluate the hypothesis that pathological changes in astrocytes are central to the pathogenesis of AD. We also examine the current tools available to assess astrocytic calcium signaling in the living murine brain as it has an important role in the complex interaction between astrocytes, neurons and the vasculature. Furthermore, we discuss the altered function of astrocytes in their interaction with neurons in the preservation of glutamate homeostasis and additionally address the role of astrocytes at the vascular interface and their contribution to functional hyperemia within the living murine brain in health and in AD.

## Introduction

Recent global estimates indicate that over 46 million individuals are living with dementia (Prince, 2017) and current clinical therapies are unable to slow progressive neurodegeneration. It is expected that by the year 2050, over 131 million people will have dementia (Prince, 2017). Alzheimer’s disease (AD) is the most prevalent form of dementia and aging is the strongest risk factor and driver of disease progression (De Strooper and Karran, 2016). Synaptic dysfunction is an important neuropathological correlate of cognitive impairment in individuals with AD (Terry et al., 1991; DeKosky et al., 1996; Serrano-Pozo et al., 2011; Selkoe and Hardy, 2016) but causative mechanisms remain elusive. An unbiased stereological analyses of a large cohort of human AD brain tissues asserted that the number of *reactive* astrocytes increased linearly with increasing disease severity and amyloid plaque burden reached a plateau early in the pathogenesis of AD (Serrano-Pozo et al., 2011). *Reactive* astrocytes undergo a complex cascade of morphological alterations that include hypertrophy and the upregulation of cytoskeleton proteins such as glial fibrillary acidic protein (GFAP) and vimentin, which may interfere with the multitude of complex physiological homeostatic functions conducted by astrocytes and contribute to the evolution of AD. Indeed, position emission tomography (PET) imaging studies using the tracer ^11^C-deuterium-L-deprenyl, directed towards the monoamine oxidase B enzyme, reported that *reactive* astrocytes are present in patients at early stages of AD, particularly those patients with Mild Cognitive Impairment (MCI) (Carter et al., 2012) and that *reactive* astrocytes precede amyloid plaques in a mouse model of AD (Rodriguez-Vieitez et al., 2015).

## *In Vivo* Imaging of Astrocytes Within the Intact Murine Brain

The normal morphology of astrocytes within the murine brain consists of a central core, occupying approximately 25 percent of the volume of the entire cell, which can range from 3000–35,000 μm^3^ (Bindocci et al., 2017). The perivascular endfoot processes occupy only 3.5 percent of cellular volume and contribute to a continuous lining on the abluminal surface of cerebral vessels (McCaslin et al., 2011). Perivascular endfoot processes express a rich complement of membrane molecules that include potassium and water channels that are important for ionic and osmotic regulation (Yang et al., 2011). The remaining cellular volume is occupied by approximately 3–9 processes radiating from the soma, each with an estimated length ranging between 5–64 μm, in addition to a peripheral region densely populated by very fine and optically unresolvable processes (Bindocci et al., 2017). Astrocytes have been studied extensively *in vitro*, thus providing a useful proxy for astrocytes *in vivo* (Cahoy et al., 2008), however astrocytes exist within artificial experimental conditions *in vitro* and may express immune system genes not expressed by astrocytes *in vivo* (Cahoy et al., 2008). Therefore, experiments testing hypotheses pertaining to the structure and function of astrocytes in health and in AD should aim to preserve the intricate interrelations between astrocytes, neurons and the vasculature by examining astrocytic signaling *in vivo*, which permits discrimination between physiological and pharmacological effects (Khakh and McCarthy, 2015).

Two photon excitation laser scanning microscopy is especially well suited to the *in vivo* study of the morphology and function of fluorescently labeled astrocytes, throughout hundreds of microns of intact cortex within rodent brain (Denk et al., 1990; Svoboda and Yasuda, 2006). Two photon microscopy offers a tight laser focus of highly localized excitation in contrast to using either wide-field or confocal microscopy, in which a scattering induced loss of excitation photons can greatly reduce imaging depth (Svoboda and Yasuda, 2006). Furthermore, mice can be trained to become habituated to head-restraint so that they are awake during two-photon imaging, thus circumventing the need for using general anesthesia (Shih et al., 2012a).

The *in vivo* imaging of astrocytes within the living mouse brain by two-photon microscopy requires an aseptic surgical implantation of a chronic cranial window in which a portion of skull bone (0.8–12 mm in diameter; Holtmaat et al., 2009) is replaced with a coverslip, that can be tightly sealed using dental cement. The degree of surgical skill elicited during preparation of the cranial window will largely determine the quality and duration of optical clarity of the window, which has been shown to be suited for repeated imaging for at least 6 months (Hefendehl et al., 2011). Alternatively, a small area of murine skull bone (2 mm in diameter; Shih et al., 2012b) can be thinned using a microsurgical blade to a final thickness of approximately 10–20 μm (Holtmaat et al., 2009; Drew et al., 2010; Shih et al., 2012b). Additional skull polishing, cryanoacrylate adhesive and a coverslip can help stabilize the weakened skull bone and prolong optical access to thinned skull preparations, permitting repeated two-photon microscopic examination of astrocytes within the living murine cortex for up to 3 months, without need for repeated surgery (Shih et al., 2012b). The chronic cranial window and thinned skull preparation are each technically challenging but both procedures, once mastered, can be conducted without inducing astrogliosis (Drew et al., 2010; Hefendehl et al., 2011; Zhang et al., 2015).

## *In Vivo* Imaging of Astrocytic Calcium Dynamics in AD Mouse Models

**Figure 1:**
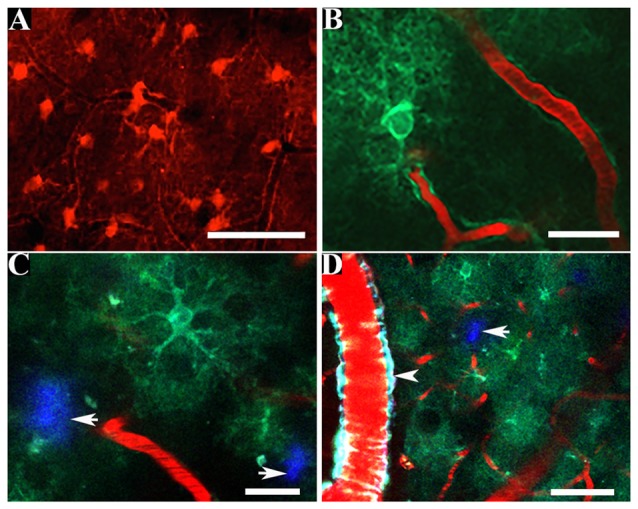
*In vivo* multiphoton imaging of astrocytic structure within the living mouse brain. Astrocytes fluorescently labeled by topical application of sulforhodamine 101 (SR-101) reveal astrocytic somas, proximal processes and perivascular endfoot processes **(A)** whereas the intracortical injection of a virally transduced genetically encoded calcium indicator (GECI) can be used to reveal the finer astrocytic processes *in vivo* **(B)**. GECIs can be transduced by astrocytes within the amyloid plaque containing brain of a murine model of Alzheimer’s disease **(C,D)**. Amyloid plaques are fluorescently labeled by methoxy-X04 (white arrows; **C,D**). The presence of a vessel affected by cerebral amyloid angiopathy (CAA) (positive for methoxy-X04) is clearly visible (white arrowhead; **D**). Scale bar = 50 μm **(A,D)**, 20 μm **(B,C)**.

The physiological function of astrocytes is intricately coupled with a multitude of spontaneous oscillations in the concentration of intracellular calcium ions that have considerable spatial and temporal heterogeneity throughout the astrocyte (see Supplementary Figure S1; Bazargani and Attwell, 2016; Bindocci et al., 2017). It remains incompletely understood how the spontaneous calcium activity of astrocytes is decoded into local and global neurovascular function in brain health and in AD (Bazargani and Attwell, 2016; Bindocci et al., 2017). Fluorescent calcium indicators such as fura-2, fluo-3, fluo-4 and Oregon Green 488 BAPTA-1 (OGB-1) permit observation of the complex and asynchronous calcium signaling occurring within and between murine astrocytes *in vitro* (Cornell-Bell et al., 1990; Glaum et al., 1990; Jensen and Chiu, 1990; Charles et al., 1991), *in situ* (Dani et al., 1992) and *in vivo* (Hirase et al., 2004; Kuchibhotla et al., 2009). In particular, the acetoxymethyl ester form of OGB-1 (OGB-1AM) can be delivered by pressurized ejection from a micropipette at a depth of approximately 100–200 μm below the murine cortical surface (Stosiek et al., 2003). Concomitant use of the fluorescent dye, sulforhodamine 101 (SR-101) provides a method for discriminating astrocytes from other cells within the living mouse cortex because the multi-cell bolus loading of calcium sensitive dyes results in a universal labeling of all cells (Nimmerjahn et al., 2004). SR-101 can be delivered to astrocytes within the living brain of mice under anesthesia by either carefully perforating the dura, ensuring minimal damage to cortical blood vessels, or by intracortical injection using a micropipette (Nimmerjahn and Helmchen, 2012). The SR-101 dye is actively taken up by astrocytes and diffusional spread occurs through the astrocytic syncytium via astrocytic gap junctions, permitting *in vivo* imaging over several hours (see Figure 1A). Experimental protocols requiring chronic *in vivo* imaging of astrocytes over days or weeks can be technically challenging considering the requirement to deliver SR-101 into each mouse brain prior to each imaging session (Nimmerjahn et al., 2004; Nimmerjahn and Helmchen, 2012), which involves the re-opening of the cranial window.

The topical application of SR-101 onto the brain of 5–9 months old APPswe/PS1dE9 (APP/PS1) transgenic AD mice (Jankowsky et al., 2001) with subsequent *in vivo* imaging by two-photon microscopy revealed that astrocytes retain their exclusive and highly organized domains in the presence of amyloid plaques, thus supporting the hypothesis that amyloid plaques do not exert chemotactic effects on astrocytes within the living murine brain (Galea et al., 2015). Additionally, a concomitant multi-cell bolus loading of OGB-1AM with a subsequent intracortical injection of SR-101 following surgical implantation of a cranial window provided optical access to measure calcium activity in astrocytes within the somatosensory cortex of the living brain of APP/PS1 transgenic and age-matched wild-type mice (Kuchibhotla et al., 2009). This study demonstrated that in the living brain of 6–8 months old APP/PS1 transgenic mice, which progressively accumulates amyloid plaques from 4–5 months of age (Jankowsky et al., 2001; Garcia-Alloza et al., 2006), there is a >80% increase in spontaneous intracellular calcium activity within astrocytes when compared to age-matched wild-type mice (Kuchibhotla et al., 2009). Additionally, astrocytes within amyloid plaque containing brain regions in the intact brain of APP/PS1 transgenic mice exhibit spontaneous intercellular propagating calcium waves that occur independently of neuronal activity (Kuchibhotla et al., 2009). *Reactive* astrocytes associated with amyloid plaques exhibit an increased expression of the purinergic P2Y1 receptor (P2Y1R; Delekate et al., 2014) and transgenic mice that overexpress the P2Y1R exhibit spontaneous intercellular propagating calcium waves with increased amplitude that are independent of local neuronal activity (Shigetomi et al., 2018). These findings suggest that astrocytes spatially associated with amyloid plaques may propagate global astrocytic calcium waves that are at least partly mediated by purinergic signaling and occur independently of synaptic transmission.

The finer perisynaptic processes of astrocytes (see Figure 1B) are the predominant intracellular sites of initiation for the spontaneous astrocytic calcium waves and are thus important for interrogating the interrelationship between astrocytic calcium signaling and synaptic function in health and in the course of AD (Shigetomi et al., 2018; see Figure 1C). A Sholl morphological analyses of astrocytes bulk loaded with the commonly used calcium sensitive dyes reported that the detection of astrocytic calcium activity was limited to the soma and proximal processes to a radial distance of approximately 20 μm, with no detection of the calcium dynamics in the finer processes (Reeves et al., 2011). Genetically encoded calcium indicators (GECIs), such as the Fluorescence Resonance Energy Transfer (FRET)-based Cameleons (Miyawaki et al., 1997; Nagai et al., 2004) and the *single* wavelength GCaMP series (Nakai et al., 2001) offer a uniform, stable and specific fluorescent labeling of astrocytes and their processes to a radial distance of approximately 50 μm, thus resolving the finer perisynaptic processes (Shigetomi et al., 2013).

Cameleon (yellow cameleon; yc2.60 or yc3.60) consist of a calmodulin moiety fused to a M13 calmodulin binding peptide and two fluorescent proteins (cyan-yellow FRET pair) (Miyawaki et al., 1997). The binding of free calcium to calmodulin results in an intramolecular rearrangement which aids the energy transfer between both fluorescent proteins leading to a change in fluorescence ratio (Pérez Koldenkova and Nagai, 2013). These GECIs are known as ratiometric because the ratio between the fluorescence emission intensities of both fluorescent proteins reflects the concentration of free cytosolic calcium (Pérez Koldenkova and Nagai, 2013). The GCaMP series (GCaMP3, GCaMP5, GCaMP6) comprise an intensiometric chimeric construct consisting of a circularly permutated green fluorescent protein that exhibits an altered emission fluorescence intensity upon the binding of free calcium (Nakai et al., 2001). Thus, they are useful for measuring dynamic changes in intracellular calcium but not for quantitative measures of concentration. The astrocytic-specific expression of GECIs can be achieved by the transduction of an adeno-associated virus of the 2/5 serotype with a promoter such as gfaABC_1_D (Shigetomi et al., 2013) that can be injected into the brain region of interest of anesthetized mice (Jiang et al., 2014). GECIs can also be targeted to the astrocytic plasma membrane by an additional membrane-tethering domain thus increasing exposure to subcellular calcium dynamics resulting in the detection of a significantly greater number of calcium signals within astrocytic somas and processes when compared to the bulk loading of Fluo-4 AM (Shigetomi et al., 2010, 2013).

## *In Vivo* Imaging of Astrocytes in Functional Hyperemia

Physiological brain function is heavily dependent upon a continuous and uninterrupted blood supply that can adapt to meet the metabolic needs of active brain regions (Girouard and Iadecola, 2006). This complex phenomenon, termed functional hyperemia, involves a sophisticated spatiotemporally correlated interplay between neurons, astrocytes and local hemodynamic responses, mediated by smooth muscle cells and endothelial cells (Zonta et al., 2003; Girouard and Iadecola, 2006; MacVicar and Newman, 2015; Tarantini et al., 2017). Impaired functional hyperemia is evident in individuals with AD at mild severity (Janik et al., 2016) and in transgenic AD mice (Niwa et al., 2000; Park et al., 2004). Therefore, deciphering the functional relationship between neurons, astrocytes and the vasculature is critical to further understanding of the pathogenesis of AD. Acute brain slices have provided useful demonstration of evoked synaptic release of glutamate which triggers propagating oscillations of intracellular calcium ions in astrocytic endfeet with temporally correlated release of vasoactive metabolites that elicit hemodynamic responses (Zonta et al., 2003; Mulligan and MacVicar, 2004; Bazargani and Attwell, 2016). However, acute brain slices are devoid of intraluminal flow required for removing vasoactive agents and for preserving myogenic tone, thus the complexities underlying the role of astrocytes in functional hyperemia may best be resolved *in vivo* using two photon microscopy, independently of the use of anesthesia (Zonta et al., 2003; Girouard and Iadecola, 2006; Takano et al., 2006; Bazargani and Attwell, 2016).

Sensory stimulation of the living murine barrel cortex *in vivo* evokes calcium signaling in neurons and astrocytes with instantaneous hemodynamic responses that correlate with elevated calcium activity in astrocytic somas and perivascular endfeet but not within parenchymal astrocytic processes or neuronal somas (Lind et al., 2013). Independent and isolated calcium responses in astrocytic perivascular endfeet, elicited by sensory stimulation in mouse brain *in vivo*, was most closely associated with the vasodilatory hemodynamic responses to neuronal activity (Lind et al., 2018). Additionally, evoked perivascular endfeet calcium responses *in vivo* was similar at penetrating arterioles and capillaries and the temporal dynamics were similar when labeled with either OGB-1AM/SR-101 or the GECI GCaMP6f (Lind et al., 2018). An *in vivo* examination of the spatiotemporal oscillations in calcium signaling in astrocytic perivascular endfeet concomitantly with changes in cerebral vascular diameters and/or cerebral blood flow requires a robust fluorescent labeling of perivascular endfeet that can be difficult to achieve when using the bulk loading of calcium sensitive indicators, such as OGB-1AM (Delekate et al., 2014).

## *In Vivo* Imaging of Glutamate Dynamics in AD

Glutamatergic signaling dysfunction is a significant pathological component within the human AD brain (Jacob et al., 2007) and in transgenic murine models of AD (Masliah et al., 2000; Hefendehl et al., 2016). Optimal physiological glutamatergic signaling is mediated by the interplay between neurons and astrocytes thus protecting post-synaptic glutamate receptors from chronic overstimulation, which can lead to neuronal injury and death. The tightly controlled calcium-dependent release of glutamate from neurons activates post-synaptic N-methyl-D-aspartate (NMDA) and alpha-amino-3-hydroxy-5-methyl-4-isoxazolepropionic acid (AMPA) glutamate receptors to increase intracellular calcium and sodium and evoke action potentials (Lin et al., 2012). Astrocytes predominantly clear glutamate via functional excitatory amino-acid transporters (EAATs) and convert glutamate to glutamine for neurons which convert glutamine to glutamate for storage and release from synaptic vesicles (Lin et al., 2012). Significant gene and protein alterations of the NMDA and AMPA glutamate receptors and EAAT1 and EAAT2 glutamate transporters was detected in the hippocampus and frontal cortex of human AD brain tissues (Jacob et al., 2007). Immunohistochemical analyses of human AD brain tissues reported loss of astrocytic EAAT receptor expression in close proximity to amyloid plaques (Jacob et al., 2007) with a trend for decreased immunoreactivity of EAAT2 with increasing Braak stage, which was inversely correlated with increasing GFAP immunoreactivity in astrocytes with disease duration (Simpson et al., 2010).

Spontaneous or sensory evoked local glutamate dynamics within the intact murine brain can be evaluated using two-photon imaging of the genetically encoded intensity-based glutamate sensing fluorescent reporter (iGluSnFR; Marvin et al., 2013; Hefendehl et al., 2016), which has greater temporal resolution than the microdialysis technique (Marvin et al., 2013; Cifuentes Castro et al., 2014). Multiphoton microscopic examination of the iGluSnFR within the intact brain of APP/PS1 transgenic mice (Radde et al., 2006) reported that the plaque microenvironment had aberrant spontaneous fluctuations in the local glutamate concentration, reduced glutamate clearance following sensory stimulation and loss of the murine glutamate receptor-1 (GLT-1/EAAT2; Hefendehl et al., 2016). The selective deletion of GLT-1 in murine astrocytes resulted in spontaneous seizures and premature death, which was not evident following the selective deletion of GLT-1 in neurons (Petr et al., 2015). The administration of the beta-lactam antibiotic, ceftriaxone to APP/PS1 mice partly restored GLT-1 expression and improved the glutamatergic activity *in vivo* (Hefendehl et al., 2016). This suggests that aberrant astrocytic function contributes to the toxic microenvironment of amyloid plaques in AD and may be amenable to pharmacological intervention.

## Future Perspectives

In 1909, Santiago Ramón y Cajal proposed that the physiological role of glia was incompletely understood because physiologists did not have the necessary tools to examine glial function (Somjen, 1988; Barres et al., 1990). The advent of new tools for the *in vivo* imaging of astrocytes within the intact murine brain may accelerate the generation of new hypotheses for elucidating the roles of astrocytes within normal physiology and in the pathogenesis of AD. The segmental accumulation of amyloid peptide in the walls of cerebral arteries and capillaries is termed cerebral amyloid angiopathy (CAA; see Figure 1D) and is associated with intracerebral hemorrhages and degenerative morphological changes to amyloid peptide laden vessels (Biffi and Greenberg, 2011). Post-mortem confirmed AD brain tissues exhibit CAA in >90% of cases (Kalaria and Ballard, 1999; Jellinger, 2002) and astrocytic perivascular endfeet that are spatially associated with vessels moderately affected by CAA exhibit reduced expression of the bidirectional water channel, aquaporin-4 (AQP4), in the medial temporal lobe of human AD brain tissues (Wilcock et al., 2009). Transmission electron microscopic examination of brain sections from the Tg-ArcSwe transgenic mouse model of AD (Lord et al., 2006) showed close spatial association between astrocytic perivascular endfeet and amyloid peptide on cerebral vessels, which disrupts the close interaction between astrocytic endfeet and endothelium (Yang et al., 2011). Correspondingly, immunohistochemical analysis of brain sections from the arcAβ transgenic mouse model of AD (Knobloch et al., 2007) showed the retraction and loss of astrocytic perivascular endfoot processes at amyloid peptide laden vessels and parenchymal amyloid plaques at early stages of pathology (Merlini et al., 2011). The development of new probes to specifically target AQP4 *in vivo* would permit the discrimination of perivascular endfeet from astrocytic processes *in vivo*. Additionally, the combined use of a specific fluorescent probe for AQP4 with the systemic administration of methoxy-X04 (Klunk et al., 2002) may permit a longitudinal examination of the spatial association between the morphology of astrocytic perivascular endfeet and CAA within the intact brain of murine models of AD. The development of fluorescent probes to target astrocytic cytoskeletal proteins such as GFAP and/or vimentin would permit the *reactive* phenotype of astrocytes and their spatial association with amyloid plaques and CAA to be examined longitudinally within murine models of AD and should prove to be powerful. The *reactive* astrocytic phenotype is heterogeneous and the nature of the cerebral insult can determine whether astrocytes adopt a neurotoxic *reactive* phenotype (termed A1) or adopt the A2 reactive subtype, which may promote neuronal health (Zamanian et al., 2012; Liddelow et al., 2017). The development of fluorescent labeling tools capable of discriminating between *reactive* subtypes may provide an excellent opportunity to assess the *reactive* phenotype in astrocytes in AD murine models so that the pharmacological effects of modulating astrocytes may be assessed longitudinally. A previous *in vivo* two-photon microscopy study demonstrated that the hyperactive calcium signaling phenotype exhibited by astrocytes within the cortex of APP/PS1 mice is amenable to pharmacological modulation (Delekate et al., 2014). The frequency of astrocytes exhibiting hyperactive calcium signaling was reduced following topical application of antagonists of purinergic signaling and increased by elevating the concentration of ADP nucleotides within the cortex of anesthetized APP/PS1 mice (Delekate et al., 2014). Additionally, an *in vivo* two photon microscopy study that examined the effects of chronic antagonism of the purinergic signaling within the intact brain of APP/PS1 mice reported a reduction in the fraction of hyperactive astrocytes associated with preservation of synaptic structure and function (Reichenbach et al., 2018). These original research findings collectively support the current relevance of using an *in vivo* two-photon imaging-based interrogation of astrocytes in AD which enables the testing of candidate therapies directed towards protecting the integrity of the neurovascular unit against the pathological impact of AD.

## Author Contributions

PK, EH, SH and BB prepared the manuscript and figures.

## Conflict of Interest Statement

The authors declare that the research was conducted in the absence of any commercial or financial relationships that could be construed as a potential conflict of interest.

## Supplementary Material

FIGURE S1 *In vivo* multiphoton imaging of the spatiotemporal intracellular astrocytic calcium dynamics within the living brain of a mouse model of Alzheimer’s disease (AD). The astrocyte expresses the virally transduced genetically encoded calcium indicator (GECI) YC3.6 delivered by intracortical injection of the AAV followed by surgical implantation of a cranial window. The 10-min time course video demonstrates spontaneous oscillations in calcium throughout an astrocyte with the ratiometric changes in calcium concentration pseudo colored from blues to red.
