# Supplementary figures and images for: In Vivo Two Photon Imaging of Astrocytic Structure and Function in Alzheimer’s Disease
Source: Front Aging Neurosci. 2018 Jul 19;10:219. doi: 10.3389/fnagi.2018.00219 (PMC6060286; doi:10.3389/fnagi.2018.00219)

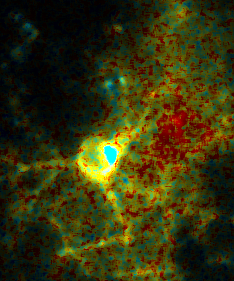

Supplement: FIGURE S1 — In vivo multiphoton imaging of the spatiotemporal intracellular astrocytic calcium dynamics within the living brain of a mouse model of Alzheimer’s disease (AD). The astrocyte expresses the virally transduced genetically encoded calcium indicator (GECI) YC3.6 delivered by intracortical injection of the AAV followed by surgical implantation of a cranial window. The 10-min time course video demonstrates spontaneous oscillations in calcium throughout an astrocyte with the ratiometric changes in calcium concentration pseudo colored from blues to red. [file Image_1.tif]
